# High Fat Feeding Induces Hepatic Fatty Acid Elongation in Mice

**DOI:** 10.1371/journal.pone.0006066

**Published:** 2009-06-26

**Authors:** Maaike H. Oosterveer, Theo H. van Dijk, Uwe J. F. Tietge, Theo Boer, Rick Havinga, Frans Stellaard, Albert K. Groen, Folkert Kuipers, Dirk-Jan Reijngoud

**Affiliations:** 1 Department of Pediatrics, Center for Liver Digestive and Metabolic Diseases, University Medical Center Groningen, University of Groningen, Groningen, The Netherlands; 2 Department of Laboratory Medicine, Center for Liver Digestive and Metabolic Diseases, University Medical Center Groningen, University of Groningen, Groningen, The Netherlands; AgroParisTech, France

## Abstract

**Background:**

High-fat diets promote hepatic lipid accumulation. Paradoxically, these diets also induce lipogenic gene expression in rodent liver. Whether high expression of these genes actually results in an increased flux through the *de novo* lipogenic pathway *in vivo* has not been demonstrated.

**Methodology/Principal Findings:**

To interrogate this apparent paradox, we have quantified *de novo* lipogenesis in C57Bl/6J mice fed either chow, a high-fat or a n-3 polyunsaturated fatty acid (PUFA)-enriched high-fat diet. A novel approach based on mass isotopomer distribution analysis (MIDA) following 1-^13^C acetate infusion was applied to simultaneously determine *de novo* lipogenesis, fatty acid elongation as well as cholesterol synthesis. Furthermore, we measured very low density lipoprotein-triglyceride (VLDL-TG) production rates. High-fat feeding promoted hepatic lipid accumulation and induced the expression of lipogenic and cholesterogenic genes compared to chow-fed mice: induction of gene expression was found to translate into increased oleate synthesis. Interestingly, this higher lipogenic flux (+74 µg/g/h for oleic acid) in mice fed the high-fat diet was mainly due to an increased hepatic elongation of unlabeled palmitate (+66 µg/g/h) rather than to elongation of *de novo* synthesized palmitate. In addition, fractional cholesterol synthesis was increased, *i.e.* 5.8±0.4% *vs.* 8.1±0.6% for control and high fat-fed animals, respectively. Hepatic VLDL-TG production was not affected by high-fat feeding. Partial replacement of saturated fat by fish oil completely reversed the lipogenic effects of high-fat feeding: hepatic lipogenic and cholesterogenic gene expression levels as well as fatty acid and cholesterol synthesis rates were normalized.

**Conclusions/Significance:**

High-fat feeding induces hepatic fatty acid synthesis in mice, by chain elongation and subsequent desaturation rather than *de novo* synthesis, while VLDL-TG output remains unaffected. Suppression of lipogenic fluxes by fish oil prevents from high fat diet-induced hepatic steatosis in mice.

## Introduction

Non-alcoholic fatty liver disease (NAFLD) is one of the hallmarks of the metabolic syndrome and is strongly associated with obesity and insulin resistance [1]. NAFLD is characterized by the accumulation of hepatic triglycerides (TGs) resulting from an imbalance between uptake, synthesis, export and oxidation of fatty acids [2]. NAFLD may progress to non-alcoholic steatohepatitis (NASH) in response to a ‘second hit’ [3]. Although high fat diets consistently induce hepatic steatosis and insulin resistance in humans and laboratory animals [4]–[6], the mechanisms underlying this high fat diet-induced lipid accumulation are largely unknown.

Interestingly, high-fat feeding has been reported to result in a paradoxical increase in the expression of lipogenic genes in mouse liver. This was suggested to be mediated via peroxisome proliferator activated receptor gamma co-activator 1 beta (PGC-1ß) co-activation of the lipogenic transcription factor sterol regulatory element binding protein 1c (SREBP-1c) [7]. Ablation or suppression of critical genes controlling hepatic lipogenesis [8]–[11] counteracts the development of hepatic steatosis in animals receiving high-fat diets. Furthermore, partial substitution of the fat within a high-fat diet for fish oil, a source of n-3 polyunsaturated fatty acids (PUFA), abrogates hepatic lipid accumulation [6]. An inhibition of the activity of lipogenic transcription factors and the subsequent suppression of their target genes by n-3 PUFA [12] is considered to contribute to the protective effects of fish oil. Although these observations suggest that the activity of lipogenic enzymes is related to the degree of high fat diet-induced hepatic steatosis, an increased *de novo* fatty acid synthesis appears counterintuitive under conditions of a high dietary fatty acid load.

Accurate quantification of fatty acid synthesis and its contribution to hepatic lipid content has not been reported. Furthermore, the relative contributions of *de novo* lipogenesis (*i.e.*, synthesis from acetyl-CoA moieties) and chain elongation of fatty acids to hepatic lipid synthesis *in vivo* in mice are currently unknown. In addition, the relationships between high-fat feeding, fatty acid synthesis and hepatic VLDL-TG production are of particular interest because of the reported alterations in plasma (VLDL)-TG levels following *Srebp1* and *Pgc-1ß* overexpression and knockdown in mice [7], [13]. We therefore determined *in vivo* rates of fatty acid and cholesterol synthesis in relation to VLDL-TG production rates in mice fed low-fat laboratory chow, a high-fat diet containing beef fat (rich in saturated fat), or a diet in which part of the beef fat was replaced by fish oil. A novel approach based on ^13^C-acetate incorporation followed by mass isotopomer distribution analysis (MIDA) enabled us to quantify the relative contribution of the *de novo* lipogenic pathway as well as chain elongation of *de novo* synthesized and pre-existing palmitate (C16:0) to stearic acid (C18:0) synthesis and its subsequent desaturation to oleic (C18:1 n-9) acid. We found that high-fat feeding indeed increased oleic acid synthesis, however this was mainly due to chain elongation of pre-existing palmitate rather than to an increase in *de novo* lipogenesis. Cholesterol synthesis was also increased while VLDL-TG secretion remained unaffected. These metabolic changes contributed to hepatic TG and cholesterol-ester accumulation. Fish oil reduced both *de novo* lipogenesis and chain elongation and normalized hepatic lipid contents.

## Results

### High-fat feeding induces hepatic lipogenic gene expression in parallel to lipid accumulation

**Figure 1:**
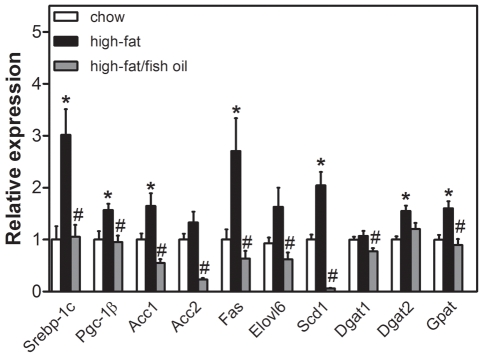
Hepatic lipogenic gene expressions. Data were calculated relative to the expression of *18S* and normalized for expression levels of control mice on chow. *Srebp-1c*, sterol regulatory element binding protein 1c; *Pgc-1β*, peroxisome proliferator activated receptor gamma co-activator 1 beta; *Acc*, acetyl-CoA carboxylase; *Fas*, fatty acid synthase; *Elovl6*, fatty acid elongase 6; *Scd1*, stearoyl-CoA desaturase 1; *Dgat*, diacylglycerol acyltransferase; *Gpat*, glycerol-3-phosphate acyltransferase. White bars represent chow diet; black bars represent high-fat diet and grey bars represent high-fat/fish oil diet. Values are given as means±SEM for *n* = 6/7; * *p*<0.05 high-fat *vs.* chow; # *p*<0.05 high-fat/fish oil *vs.* high-fat.

**Figure 2:**
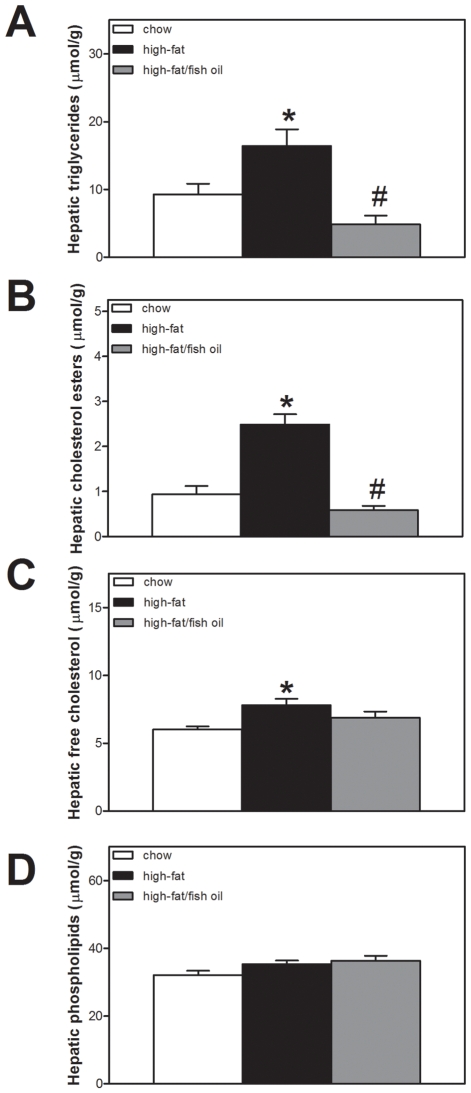
Hepatic lipid content. A, Hepatic triglyceride content. B, Hepatic cholesterol ester content. C, Hepatic free cholesterol content. D, Hepatic phospholipid content. White bars represent chow diet; black bars represent high-fat diet and grey bars represent high-fat/fish oil diet. Values are given as means±SEM for *n* = 6/7; * *p*<0.05 high-fat *vs.* chow; # *p*<0.05 high-fat/fish oil *vs.* high-fat.

Mice fed the high-fat and high-fat/fish oil diet had higher caloric intakes (chow, 12.3±0.1; high-fat, 17.1±0.8, high-fat/fish oil 16.8±1.0 kcal/day, *p*<0.05 high-fat *vs.* chow). At the end of the dietary period, their body weight were increased compared to that of chow-fed animals (chow, 28.2±0.4; high-fat, 30.5±0.8; high-fat/fish oil, 33.4±0.8 g, *p*<0.05 high-fat *vs.* chow and high-fat/fish oil *vs.* high-fat). This was due to an increased fat mass (chow, 2.1±0.1; high-fat, 6.3±0.5; high-fat/fish oil, 7.6±0.4% of total body weight, *p*<0.05 high-fat *vs.* chow). Expression of genes encoding enzymes involved in hepatic fatty acid (*Acc*, *Fas*, *Scd1*, *Elovl6*) and TG synthesis (*Dgat*, *Gpat*) was induced in mice fed the high-fat diet compared to animals receiving chow. Furthermore, *Srebp-1c* and *Pgc-1β* expression was higher in these mice as compared to controls (Figure 1). The increase in lipogenic gene expression was associated with increases in hepatic TG (chow, 9.2±1.6; high-fat, 16.4±2.4 µmol/g, *p*<0.05) cholesterol ester (chow, 0.9±0.2; high-fat, 2.5±0.2 µmol/g, *p*<0.05) and free cholesterol (chow, 6.0±0.2; high-fat, 7.8±0.5 µmol/g, *p*<0.05) contents in mice fed the high-fat diet. Partial replacement of the saturated fat within the high-fat diet by n-3 PUFA strongly suppressed hepatic lipogenic gene expression (Figure 1) and normalized hepatic TG and cholesterol ester contents (Figure 2A and 2B) to values observed in chow-fed mice. Hepatic phospholipid contents (Figure 2C and 2D) and liver weights (chow, 0.96±0.07; high-fat, 1.08±0.04; high-fat/fish oil, 1.08±0.03 g) were similar in all mice. Hepatic fatty acid profiles are shown in Table 1. The total amount of fatty acids was increased in mice fed the high-fat diet compared to chow-fed controls due to accumulation of TG and cholesterol esters. In general, the contribution of mono-unsaturated fatty acids was increased by high-fat feeding and desaturation indices were consequently increased. Fish oil normalized hepatic mono-unsaturated fatty acid content and the desaturation indices.

**Table 1:** Fatty acid composition of experimental diets and livers.

	chow	high-fat	high-fat/fish oil
***Diet (mg/g)***			
C14:0	0.5	12.2	16.1
C16:0	8.4	92.5	79.5
C16:1	0.7	11.5	18.0
C18:0	3.7	76.3	50.5
C18:1	13.7	133.2	101.0
C18:2	16.9	11.5	9.7
C18:3	1.9	2.9	15.2
C20–22	0.4	4.0	53.3
C16 desaturation index	0.08	0.12	0.18
C18 desaturation index	3.7	1.7	2.0
***Liver (mg/g)***			
C14:0	0.1±0.0	0.2±0.0*	0.1±0.0#
C16:0	7.7±0.6	9.0±0.3	8.2±0.6
C16:1	0.6±0.1	1.0±0.1*	0.4±0.1#
C18:0	4.1±0.3	4.8±0.2	5.0±0.2
C18:1	6.0±0.4	16.9±2.1*	6.1±0.6#
C18:2	7.0±0.7	2.8±0.2*	2.9±0.3
C18:3	0.3±0.0	0.2±0.0*	0.2±0.0
C20–22	8.4±0.5	9.2±0.5	11.1±0.6#
C16 desaturation index	0.07±0.00	0.11±0.01*	0.05±0.00#
C18 desaturation index	1.5±0.1	3.6±0.5*	1.2±0.1#

Values are given as means±SEM for *n* = 6/7.

* *p*<0.05 high-fat *vs.* chow.

# *p*<0.05 high-fat/fish oil *vs.* high-fat.

### High-fat feeding increases hepatic fatty acid synthesis from chain elongation

To assess whether the accumulation of hepatic lipids in response to high-fat feeding resulted from an increased *de novo* fatty acid synthesis and/or chain elongation of *de novo* synthesized versus existing palmitate, we infused [1-^13^C]-acetate and applied MIDA to the measured label distribution patterns of palmitate, stearate and oleate, assuming that label incorporation was due to *de novo* lipogenesis only. The resulting estimations of the acetyl-CoA precursor pool enrichments are shown in Table 2. Compared to chow-fed animals, acetyl-CoA pool enrichments were increased in mice fed the high-fat diet for all fatty acids analyzed. Precursor pool enrichments were similar in mice fed chow and fish oil. In general, there was a clear discrepancy in acetyl-CoA pool enrichments for palmitate (C16:0) and palmitoate (C16:1) on one hand and stearate (C18:0) and oleate (C18:1) on the other hand. The precursor pool enrichment calculated for C16-fatty acids was higher than that calculated to C18-fatty acids. This indicates that singly labeled fatty acids were high compared to triple labelled fatty acids. We interpreted this difference as a reflection of the different synthetic pathways of these fatty acids. C16-fatty acids are mainly synthesized by *de novo* lipogenesis, while C18-fatty acids result from elongation of palmitate. Palmitate can either be synthesized *de novo*, or originate from pre-existing sources. Accordingly, additional single labelled stearate is synthesized from elongation of pre-existing palmitate with a labelled acetyl-CoA moiety. This results in an excess contribution of single labelled molecules in C18-fatty acids over what could be anticipated based on the contribution of triple labelled molecules originating from elongation of *de novo* synthesized palmitate. The precursor pool enrichment, calculated by MIDA from C18-fatty acids will consequently be underestimated compared to that calculated from C16-fatty acids. Therefore, we modified the MIDA algorithms [14]–[16] to account for excess single labelled C18-fatty acids, as described in Materials and Methods.

**Table 2:** Acetyl-CoA precursor pool enrichments.

	chow	high-fat	high-fat/fish oil
C16:0	8.8±0.5	13.0±0.3*	8.4±0.3#
C16:1	10.0±0.8	13.2±0.6*	10.2±1.2#
C18:0	5.7±0.2	8.1±0.5*	4.4±0.3#
C18:1	4.5±0.3	6.7±0.7*	7.0±1.0

Values are given as means±SEM for *n* = 5–7 and expressed in percentages.

* *p*<0.05 high-fat *vs.* chow.

# *p*<0.05 high-fat/fish oil *vs.* high-fat.

**Figure 3:**
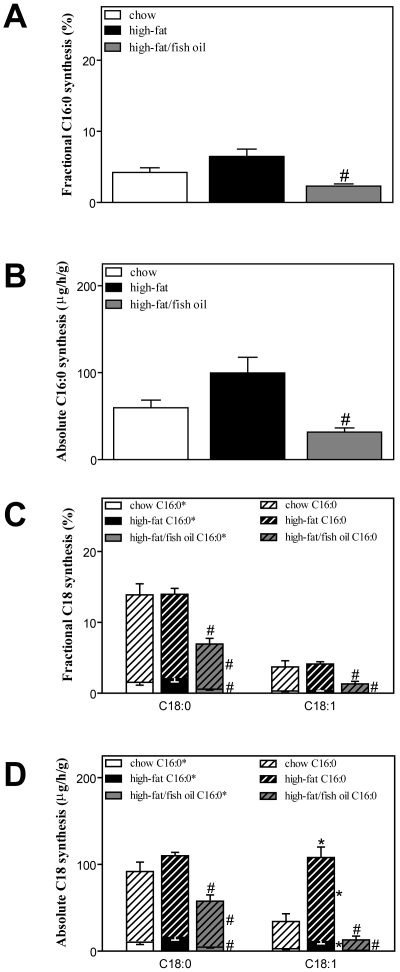
Hepatic fatty acid synthesis. A, Fractional palmitate synthesis from *de novo* lipogenesis (C16:0*). B, Absolute palmitate synthesis from *de novo* lipogenesis (C16:0*). C, Fractional stearate (C18:0) and oleate (C18:1) synthesis from elongation of *de novo* synthesized (C16:0*) and pre-existing (C16:0) palmitate. D, Absolute stearate (C18:0) and oleate (C18:1) synthesis from elongation of *de novo* synthesized (C16:0*) and pre-existing (C16:0) palmitate. White bars represent chow diet, black bars represent high-fat diet and grey bars represent high-fat/fish oil diet. Plain bars represent synthesis from elongation of *de novo* synthesized palmitate and dashed bars represent synthesis from elongation of pre-existing palmitate. Absolute synthesis rates are expressed as micrograms per gram liver per hour. Values are given as means±SEM for *n* = 5–7; * *p*<0.05 high-fat *vs.* chow; # *p*<0.05 high-fat/fish oil *vs.* high-fat.

Compared to chow-fed animals, average fractional and absolute C16:0 synthesis were increased by high-fat feeding, although the difference did not reach statistical significance (Figure 3A and 3B). High-fat feeding did not alter fractional and absolute C18:0 synthesis, and the contributions of *de novo* synthesis and chain elongation were similar compared to chow-fed mice (Figure 3C and 3D). Although high-fat feeding did not affect fractional C18:1 synthesis, the absolute synthesis by elongation of both *de novo* synthesized (+300% *vs.* chow, *p*<0.05) and pre-existing palmitate (+213% *vs.* chow, *p*<0.05) was increased as a result of the larger pool size. However, the contribution of elongation of pre-existing palmitate to the increase in C18:1 synthesis much more pronounced compared to elongation of *de novo* synthesized palmitate (89 *vs.* 11%) in high-fat fed mice. When the saturated fat was partially replaced by fish oil, fractional and absolute C16:0 synthesis were both suppressed in high fat-fed mice (−66% and −70% *vs.* high-fat, *p*<0.05, Figure 3A and 3B). Furthermore, fish oil inhibited C18:0 synthesis from elongation of both *de novo* synthesized and pre-existing palmitate (total absolute C18:0 synthesis: −48% *vs.* high-fat, *p*<0.05). Strikingly, C18:1 synthesis from elongation of *de novo* synthesized and pre-existing palmitate was almost completely abolished in fish oil-fed mice (absolute C18:1 synthesis, −91% and −89% *vs.* high-fat, *p*<0.05, Figure 3C and 3D).

### High-fat feeding increases cholesterol synthesis

**Figure 4:**
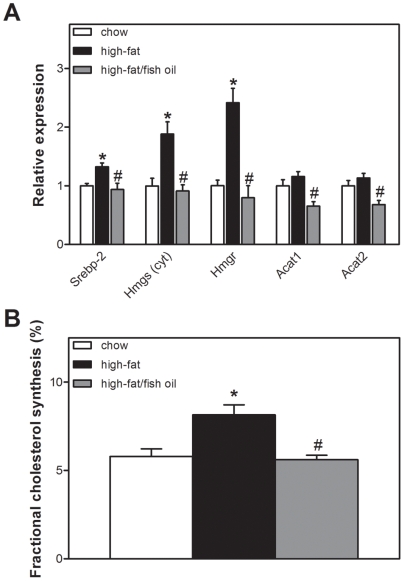
Cholesterol metabolism. A, Hepatic cholesterogenic gene expression. Cholesterogenic gene expression levels were calculated relative to the expression of *18S* and normalized for expression levels of control mice on chow. *Srebp-2*, sterol regulatory element binding protein-2; *Hmgs (cyto)*, 3-hydroxy-3-methylglutaryl-CoA synthase 1; *Hmgr*, 3-Hydroxy-3-methylglutaryl-CoA reductase; *Acat*, acyl-CoA:cholesterol acyltransferase. B, Fractional synthesis rates. White bars represent chow diet, black bars represent high-fat diet and grey bars represent high-fat/fish oil diet. Values are given as means±SEM for *n* = 5–7; * *p*<0.05 high-fat *vs.* chow; # *p*<0.05 high-fat/fish oil *vs.* high-fat.

High-fat feeding resulted in higher mRNA levels for enzymes involved in cholesterol biosynthesis (*i.e. Hmgs* and *Hmgr*) while expression of *Acat1* and *Acat2* was not affected (Figure 4A). Expression of *Srebp-2*, which encodes a transcriptional regulator of cholesterol synthesis, was also induced. We therefore determined fractional cholesterol synthesis *in vivo* following [1-^13^C]-acetate infusion and MIDA. Again, high-fat feeding increased acetyl-CoA precursor pool enrichment (chow, 5.3±0.4; high-fat, 10.2±0.8%, *p*<0.05). Moreover, high-fat feeding increased fractional cholesterol synthesis compared to chow-fed controls (chow, 5.8±0.4; high-fat, 8.1±0.6%, *p*<0.05, Figure 4B). Fish oil normalized mRNA expression of cholesterogenic genes, acetyl-CoA precursor pool enrichment (high-fat/fish oil: 6.0±0.4%, *p*<0.05 *vs.* high-fat) and fractional cholesterol synthesis (high-fat/fish oil: 5.6±0.3%, *p*<0.05 *vs.* high-fat, Figure 4B).

### High-fat feeding does not affect hepatic VLDL-TG secretion

**Figure 5:**
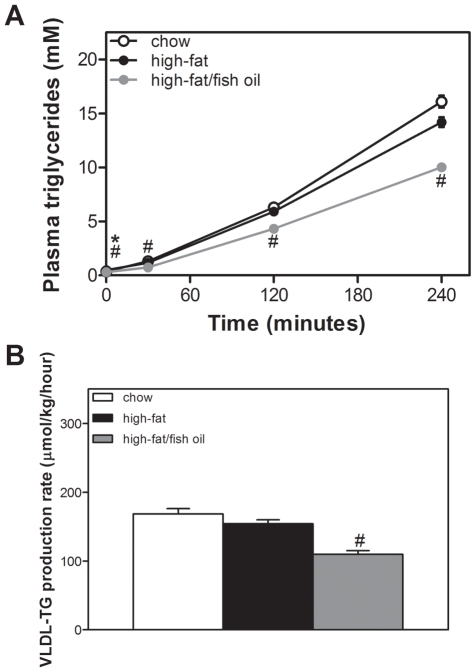
Hepatic very low density lipoprotein (VLDL) secretion. A, Plasma TG concentrations and B, VLDL-TG production rates. White bullets and bars represent chow diet, black bullets and bars represent high-fat diet and grey bullets and bars represent high-fat/fish oil diet. Values are given as means±SEM for *n* = 7–8; * *p*<0.05 high-fat *vs.* chow; # *p*<0.05 high-fat/fish oil *vs.* high-fat.

To assess whether high-fat feeding modulated hepatic lipid secretion, we determined VLDL-TG production rates. Plasma TG levels prior to Poloxamer-407 injection were somewhat higher in mice fed the high-fat diet compared to animals fed chow and lower in fish oil-fed mice (chow, 0.4±0.0; high-fat, 0.5±0.0; high-fat/fish oil 0.3±0.0 mmol/L, *p*<0.05 chow *vs.* high-fat, high-fat *vs.* high-fat/fish oil, Figure 5A). High-fat feeding resulted in a slight statistically non-significant reduction in hepatic VLDL-TG production compared to chow-fed animals (chow, 168±8; high-fat 154±6 µmol/kg/hour). In addition, the relative TG content of VLDL was decreased at the expense of phospholipids in mice fed the high-fat diet (Table 3). Calculated size of nascent VLDL was reduced by high-fat feeding and Western blot analysis revealed that high-fat feeding increased the relative amount of apoB48-containing VLDL particles compared to mice fed chow (Table 3). Fish oil suppressed hepatic VLDL-TG production (110±5 µmol/kg/hour, *p*<0.05 *vs.* high-fat, Figure 5A and 5B) and partially normalized the composition and size of the nascent VLDL particles to values observed in chow-fed animals (Table 3). In addition, fish oil partially restored the balance between apoB48 and apoB100-containing particles.

**Table 3:** VLDL composition and calculated size.

	chow	high-fat	high-fat/fish oil
Triglycerides (mol%)	76.8±0.3	68.0±1.0*	73.1±0.4#
Phospholipids (mol%)	17.6±0.4	26.7±1.0*	19.0±0.4#
Cholesterol (mol%)	5.6±0.3	5.3±0.2	7.6±0.2#
Particle diameter (nm)	71.6±1.3	48.8±1.7*	64.2±1.2#
Particle volume (10^5^ nm^3^)	1.9±0.1	0.6±0.1*	1.4±0.1#
ApoB48 (%)	80±5	92±1	86±2#
ApoB100 (%)	20±5	8±1	14±2#

Values are given as means±SEM for *n* = 7–8 for the particle composition and size data and means±SEM for *n* = 4 for the apolipoprotein B (apoB) abundance.

* *p*<0.05 high-fat *vs.* chow.

# *p*<0.05 high-fat/fish oil *vs.* high-fat.

## Discussion

The major finding of our study is that the counterintuitive induction of hepatic lipogenic genes upon high-fat feeding is paralleled by adaptive remodelling of hepatic fatty acids rather than to increased *de novo* lipogenesis. High-fat feeding also promoted cholesterol synthesis but did not stimulate VLDL-TG secretion. Consequently, TG and cholesterol esters accumulated in the livers of high fat-fed mice. Partial replacement of the saturated fat for fish oil normalized hepatic lipid content by suppressing both *de novo* lipogenesis and chain elongation as well as cholesterol synthesis.

We applied a novel approach based on *in vivo* ^13^C-acetate incorporation followed by MIDA to determine the contribution of *de novo* lipogenesis and chain elongation to the synthesis of three major hepatic fatty acids. The most commonly used method to determine hepatic lipogenesis *in vivo* in experimental animals is by quantification of the incorporation of ^3^H from ^3^H_2_O into total hepatic fatty acids. However, this method only provides a rough estimate of fractional hepatic fatty acid synthesis since ^3^H_2_O-derived label is incorporated into multiple positions in fatty acids by different metabolic pathways. Our approach provides more detailed information about the origin of the newly synthesized fatty acids. We modified the model introduced by Hellerstein and Neese [14]–[16] to determine the contributions of chain elongation of *de novo* synthesized and pre-existing palmitate to stearate and oleate synthesis. In the original model, palmitate synthesis is considered as a 8-step polymerization of acetate units. Infusion of labelled acetate *in vivo* will result in its incorporation into fatty acids. The frequency of label incorporation depends on the enrichment of the acetate pool, *i.e.* the precursor pool enrichment. The newly synthesized fatty acids will either be labelled or unlabelled. This pool of newly synthesized fatty acids is subsequently diluted in the existing pool of unlabelled fatty acids. Thus, due to the synthesis of both labelled and unlabelled fatty acids, one cannot calculate fractional fatty acid synthesis rates from the dilution of the labelled fatty acids only. Firstly, the enrichment of the acetate pool is calculated from the M_3_/M_1_ ratio, which is insensitive towards dilution. Secondly, this precursor pool enrichment is used to calculate the theoretical (*i.e.* undiluted) frequency of triple labelled fatty acids. This step in the MIDA actually also accounts for the synthesis of unlabelled fatty acids. The ratio of the theoretical frequency over the measured amplitude of a particular fatty acid mass isotopomer subsequently generates the dilution of the newly synthesized fatty acid. We applied this procedure to calculate fractional palmitate synthesis, assuming that this is solely reflects *de novo* lipogenesis. Next, we assumed that acetyl-CoA used for elongation of *de novo* synthesized and pre-existing palmitate originates from the same pool, *i.e.* that the precursor pool enrichment calculated for palmitate equals that for stearate and oleate. Finally, the mass isotopomer distribution patterns of stearate and oleate were used to calculate the contributions of elongation of *de novo* synthesized and pre-existing palmitate. Some studies have casted doubt on the homogeneity of the hepatic acetyl-CoA pool [17], [18], which could explain the difference in pool enrichments in palmitate versus cholesterol observed in the current study. However, Hellerstein *et al.*, have shown that the enrichment of the precursor pool for fatty acid synthesis is very similar to that of acetate residues in acetylated drugs [19]. Furthermore, inhomogeneous labelling of the hepatic acetyl-CoA pool has been observed at very high degrees of labelling, *i.e.*, around 70% [18]. We and others [16], [20]–[22] have avoided this issue by using protocols that result in a moderate precursor pool labelling of ∼15%.

The high fat diet-induced increase in lipogenic gene expression observed in this study, confirms earlier reports [7], [11]. We now show that the increase in lipogenic genes does not result in a significant induction of *de novo* lipogenesis, hence this pathway appears to be of minor physiological importance in the development of hepatic lipid accumulation under conditions of high-fat feeding. *De novo* synthesis of palmitic acid was not significantly increased. Furthermore, the contribution of elongation of *de novo* synthesized palmitate to absolute stearate and oleate synthesis was only minor and represented 20 and 9% of the total synthesis, respectively in chow-fed animals. A relative decrease in the contribution of *de novo* lipogenesis to hepatic TG has recently also been reported in rats fed a high-fat diet [23]. Moreover, *de novo* lipogenesis is not induced upon a short-term dietary fat challenge in human subjects [24], [25]. Palmitate synthesis from *de novo* lipogenesis may however have been overestimated in the current study because we were not able to quantify the contribution of chain elongation to the synthesis of this fatty acid. However, because of the relatively low dietary myristic acid content, we consider this contribution to be of minor importance. Chain elongation of pre-existing palmitate represented 89% of the increase in C18:1 synthesis upon high-fat feeding. Another interesting finding in our study is the observation that partial eucaloric replacement of saturated fat within the high-fat diet by fish oil completely abrogated the lipogenic effect. Partial fish oil replacement was apparently sufficient to normalize lipogenic gene expression profiles and hepatic steatosis, even under conditions of inhibited VLDL-TG secretion. The suppressive effect of n-3 PUFA on lipogenic gene expression in liver has been reported in earlier studies [12], however, the physiological effect of fish oil on *de novo* lipogenesis and chain elongation *in vivo* has not been investigated before. Our work shows that fish oil not only counteracts the increase in hepatic chain elongation, but also suppresses fatty acid synthesis via the *de novo* lipogenic pathway.

Interestingly, lipid partitioning to TG storage has recently been suggested to protect the liver from lipotoxicity [26]. Obesity and insulin resistance result in an increased flux of fatty acids from adipose tissue towards the liver [2]. If hepatic fatty acid oxidation is not sufficient to meet its influx, fatty acids may be elongated [27] and/or re-esterified to prevent their toxic accumulation [28]. On the other hand, high fat diet-induced steatosis is prevented if hepatic fatty acid influx is blocked by knockdown of the fatty acid transporter FATP5 [29]. The increased fatty acid elongation and subsequent TG synthesis upon high-fat feeding may therefore reflect a physiological buffering process. In addition, increased hepatic fatty acid content induces ACAT activity [30], [31], thereby promoting fatty acid esterification to cholesterol, as reflected by higher hepatic cholesterol-ester contents in high fat-fed mice. As a consequence, cellular free cholesterol content drops, which, in turn, provokes a compensatory SREBP-2-mediated induction of cholesterol synthesis [32], [33] that is reflected by an increased expression of cholesterogenic genes and increased cholesterol synthesis. Similar adaptive mechanisms leading to cholesterol-ester accumulation exist when mice that are unable to exert feedback-inhibition by cellular cholesterol *(i.e. Lxrα* ^−^/_−_ mice) are challenged with dietary cholesterol [34]. Thus, in response to an increased substrate supply, the liver exerts several adaptive physiological responses to prevent cytotoxic accumulation of lipid species. In the current study, the increase in oleate synthesis most likely reflects the liver's attempt to safely store saturated fatty acids as relatively harmless TGs. Palmitate was first elongated into stearate, which in turn was desaturated to oleate *via* SCD1 action. Stearate itself is only minimally present in TGs [34]. Hepatic SCD1 action actually plays a key role in the partitioning of excess lipid and enables adequate storage [26]. It has to be noted that the influx of dietary saturated fatty acids may have been limited, since we performed the experiments after a 4-hour fast. Under these conditions, fatty acid influx from adipose tissue probably dominates. Obesity and insulin resistance [2] may therefore in fact indirectly result in the increase in hepatic oleate synthesis *via* chain elongation of circulating palmitate in high fat-fed mice. It should however be noted that the lipogenic fluxes maximally accounted for 15% of the hepatic fatty acid pool. Re-esterification of circulating fatty acids therefore represents the major pathway contributing to hepatic TG disposal. The induction of protective systems upon high-fat feeding is absent in case of partial isocaloric replacement of the saturated fat by fish oil. In addition to a profound suppression of lipogenic gene expression [12], such dietary modulation apparently alters the cellular fate of fatty acids and their influx into the liver. In this respect, it should be noted that n-3 PUFA not only promote fatty acid oxidation [35], but also increase peripheral lipid clearance presumably by enhancing LPL-activity [36]–[39]. As a result, fatty acids are sequestered in extrahepatic tissues, predominantly in adipose stores. Mice fed the fish-oil containing high-fat diet indeed deposited more fat in their adipose tissue.

Despite increased substrate availability and elevated hepatic *Pgc-1β* expression [7], hepatic VLDL-TG production rate was slightly but non-significantly reduced upon high-fat feeding. Hepatic VLDL production was thus insufficient to accommodate the increase in hepatic fatty acid synthesis during high fat feeding, indicating that there was progressive steatosis in high fat-fed mice. Moreover, VLDL particle size was reduced in high fat-fed mice and the relative abundance of apoB48-associated VLDL particles was increased. The molecular mechanism underlying the decreased VLDL-TG secretion rate is not yet clear. We can, however, conclude from this study that the rate of hepatic fatty acid synthesis *per se* does not determine hepatic VLDL-TG secretion as proposed earlier [20], [21], [40] and, thus, that VLDL-TG secretion is not determined by TG availability. Other factors such as mobilization of the cytosolic TG pool and apoB availability and –fusion are therefore likely to be important controlling factors in hepatic VLDL secretion [41]–[43]. Indeed, the suppression of VLDL secretion by fish oil has been reported to be due to increased apoB degradation [44].

In summary, data reported in this study provide insight in a physiological mechanism that protects the liver from lipotoxicity under conditions of dietary fatty acid oversupply. Using a novel MIDA approach we were able to show that high-fat feeding predominantly promotes fatty acid elongation of pre-existing palmitate *in vivo*. Although high-fat feeding resulted in an induction of hepatic expression of *de novo* lipogenic genes, we did not observe a significant increase in the flux through this pathway. Furthermore, cholesterol synthesis was increased, presumably to compensate for increased cholesterol esterification. These physiological adaptations result in hepatic lipid accumulation and do not occur if fatty acid influx into the liver is arrested by partial replacement of saturated fat by fish oil. It should however be noted that the ‘harmless’ storage of excess fatty acids represents the primary event or the ‘first hit’ in the pathophysiology of NASH [3]. Consequently, such an adaptive physiological response may eventually predispose to development of liver disease, because it renders the liver more susceptible to ‘second hits’ [3].

## Materials and Methods

### Ethics Statement

Experimental procedures were approved by the Ethics Committee for Animal Experiments of the University of Groningen.

### Animals and experimental design

Male C57Bl/6J mice (Charles River, L'Arbresle Cedex, France), three months of age, were housed in a light- and temperature-controlled facility (lights on 6:30 AM–6:30 PM, 21°C). They were divided into groups and fed three different diets for six weeks. All diets were obtained from Abdiets, Woerden, The Netherlands. One group received normal laboratory chow (RMH-B), the second group received high-fat diet (beef tallow, which is rich in saturated fat) and the third group received a diet in which 42% (w/w) of the beef fat was replaced by fish oil (menhaden oil). For diet composition see Table 1. The fish oil-containing diet was refreshed three times a week to prevent oxidation. To exclude acute postprandial effects without the induction of a fasting response, mice were subjected to a short fasting period of 4 hours (6–10 AM) prior to all experiments.

### Hepatic lipid content and gene expression levels

Mice were sacrificed by cardiac puncture under isoflurane anaesthesia. Epididymal, perirenal and brown adipose fat pads were removed and weighed. Livers were quickly removed, weighed, freeze-clamped and stored at −80°C. Hepatic TG, total and free cholesterol content were analyzed using commercial available kits (Roche Diagnostics, Mannheim, Germany and Wako Chemicals, Neuss, Germany) after lipid extraction [45]. Cholesterol ester concentrations were calculated as the difference between total and free cholesterol. Hepatic phospholipid content was determined as described previously [46]. Hepatic fatty acid composition was analyzed by gas chromatography after transmethylation using C17:0 as internal standard [47]. C16 and C18 desaturation indices were calculated from the ratios between C16:1 n-7 and C16:0 and C18:1 n-7/n-9 and C18:0, respectively.

RNA was extracted from livers using Tri reagent (Sigma-Aldrich, St. Louis, MO) and converted into cDNA by a reverse transcription procedure using M-MLV and random primers according to the manufacturer's protocol (Sigma-Aldrich). For quantitative PCR (qPCR), cDNA was amplified using the appropriate primers and probes. Primer and probe sequences for *18S*, acetyl-CoA carboxylase 1/2 (*Acc1 and -2*), acyl-CoA:cholesterol acyltransferase (*Acat-1* and *-2*), diacylglycerol acyltransferase (*Dgat1* and *2*), glycerol-3-phosphate acyltransferase (*Gpat*), fatty acid synthase (*Fas*), 3-hydroxy-3-methylglutaryl-CoA reductase (*Hmgr*), stearoyl-CoA desaturase 1 (*Scd1*), sterol regulatory element binding protein 1c/2 (*Srebp-1c* and *-2*) have been published (www.LabPediatricsRug.nl). For other primer and probe sequences, see Table S1. mRNA levels were calculated relative to *18S* expression and normalized for expression levels of control mice on chow.

### Determination of de novo lipogenesis, chain elongation and cholesterol synthesis in vivo

Mice were equipped with a permanent jugular vein catheter [48] and were allowed a recovery period of 4–5 days. All infusion experiments were performed in conscious, unrestrained mice. A 0.3 mol/L sodium [1-^13^C]-acetate (99 atom %, Isotec/Sigma-Aldrich) solution was infused at a rate of 0.6 mL/hr during 6 hours. Every hour a blood sample was taken via tail bleeding on filter paper to determine fractional cholesterol synthesis rates. At the end of the infusion period, animals were sacrificed by cardiac puncture. Livers were quickly removed, freeze-clamped and stored at −80°C. Liver homogenates were prepared in PBS and C17:0 was added as internal standard. Lipids were hydrolyzed in HCl/acetonitrile (1∶22 v/v) for 45 minutes at 100°C. Fatty acids were extracted in hexane and derivatized for 15 minutes at room temperature using Br-2,3,4,5,6-pentafluorobenzyl/acetonitrile/triethanolamine (1∶6∶2 v/v). Derivatization was stopped by adding HCl and fatty acid-PFB derivatives were extracted in hexane. Total cholesterol was extracted from blood spots using ethanol/acetone (1∶1 v/v). Unesterified cholesterol from blood spots was subsequently derivatized using N,O-bis-(trimethyl)trifluoroacetamide with 1% trimethylchlorosilane at room temperature.

The fatty acid-PFB isotopomer patterns were analyzed using an Agilent 5975 series GC/MSD (Agilent Technologies, Santa Clara, CA). Gas chromatography was performed using a ZB-1 column (Phenomenex, Torrance, CA). Mass spectrometry analysis was performed by electron capture negative ionization using methane as moderating gas. Cholesterol-TMS isotopomer patterns were analyzed using a Trace MS plus GC-MS (Interscience, Breda, The Netherlands). Gas chromatography was performed using a DB-17 column (J&W Scientific, Falson, CA). Mass spectrometry analysis was performed in the electron impact mode.

The normalized mass isotopomer distributions measured by GC-MS (m_0_–m_x_) were corrected for natural abundance of ^13^C by multiple linear regression as described by Lee et al. [49] to obtain the excess fractional distribution of mass isotopomers (M_0_–M_x_) due to incorporation of the infused labelled compound, *i.e.*, [1-^13^C]-acetate. This distribution was used in mass isotopomer distribution analysis (MIDA) algorithms to calculate isotope incorporation and dilution according to Hellerstein *et al.* [14]–[16] in order to determine fractional palmitate synthesis rates. In short, incorporation of [1-^13^C]-acetate into palmitate was assumed to solely result from *de novo* lipogenesis *via* the malonyl-CoA/FAS pathway. The measured M_1_ and M_3_ isotopomers of palmitate were used to calculate the acetyl-CoA precursor pool enrichment (*p*_acetate_) and fractional palmitate synthesis (f_C16:0_).

Stearate is synthesized by chain elongation of *de novo* synthesized and/or pre-existing palmitate. The M_1_ mass isotopomer of stearate represents the sum of these two processes, while the M_3_ mass isotopomer solely results from chain elongation of labelled palmitate. The following approach was used to calculate fractional stearate and oleate synthesis. We assumed that the acetate enrichment used for elongation of palmitate equals *p*_acetate_. Stearate generated from *de novo* synthesized palmitate was consequently considered as a nonamer of acetate. Therefore, we applied MIDA algorithms using M_3_(stearate) and *p*_acetate_ to calculate fractional stearate synthesis from elongation of *de novo* synthesized palmitate (f_stearate(palmitate*)_). Total M_1_(stearate) was subsequently corrected for the contribution of single labelled stearate originating from elongation of *de novo* synthesized palmitate M_1_(stearate(palmitate*)) to obtain the contribution of single labelled stearate originating from elongation of pre-existing palmitate M_1_(stearate(palmitate)). Since we assumed that *p*_acetate_ represents the precursor pool enrichment of acetate used in elongation of pre-existing palmitate, the contribution of elongation of pre-existing palmitate to stearate synthesis f_stearate(palmitate)_ could finally be calculated.
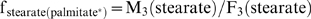
in which F_3_(stearate) equals the theoretical undiluted frequency of triple labelled stearate at *p*_acetate_.
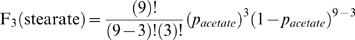
M_1_(stearate(palmitate*) is calculated according to:
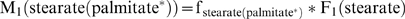
in which F_1_(stearate) equals the theoretical undiluted frequency of single labelled stearate at *p*_acetate_.
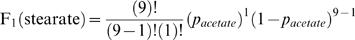
Consequently:
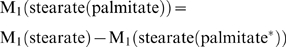
in which M_1_(stearate) represents the measured total M_1_ mass isotopomer in stearate.

And finally:
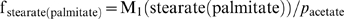



Oleate is synthesized by desaturation of stearate *via* SCD1 activity. We used the measured M_1_ and M_3_ isotopomers of oleate to calculate the fractional contributions of chain elongation of *de novo* synthesized and pre-existing palmitate to stearate as a direct precursor for oleate (f_oleate(palmitate*)_ and f_oleate(palmitate)_, respectively) using similar equations to that of stearate
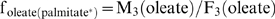
in which F_3_(oleate) equals the theoretical undiluted frequency of triple labelled oleate, calculated as for stearate using *p*_acetate_.
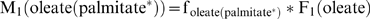
in which M_1_(oleate(palmitate*)) represents the contribution of elongation of *de novo* synthesized palmitate to M_1_(oleate) and F_1_(oleate) equals the theoretical undiluted frequency of single labelled oleate, calculated as for stearate using *p*_acetate_.
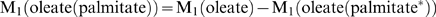
in which M_1_(oleate(palmitate)) represents the contribution of elongation of pre-existing palmitate to M_1_(oleate).
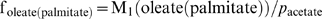



Fractional cholesterol synthesis was calculated on regular time points during isotope infusion (f_t_) by MIDA. From this, fractional cholesterol synthesis at infinite time (f_∞_) was calculated using SAAM II software (version 1.2.1 Saam Institute, University of Washington) and the following formula:
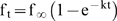
in which k represents the rate constant.

### *In vivo* VLDL-TG production

Mice were injected intraperitoneally with Poloxamer 407 (1 mg/kg body weight) as a 50 mg/mL solution in saline as previously described [50]. Blood samples were drawn by retro-orbital bleeding into heparinized tubes at 0, 30, 120, and 240 min after injection. Immediately after the last blood draw, animals were sacrificed by cardiac puncture under isoflurane anaesthesia. Blood was centrifuged (10 minutes, 4000×g) to obtain plasma. Plasma TG levels and TG production rates were determined as described [50]. Nascent VLDL (d<1.006) was isolated from the final plasma sample of each animal using a Optima TM LX tabletop ultracentrifuge (Beckman Instruments Inc., Palo Alto, CA) at 108,000 rpm for 125 minutes.

### VLDL composition and particle size

VLDL-TG and cholesterol contents were determined as described [50]. Phospholipid content was determined using a commercial kit (Wako Chemicals). VLDL particle diameter was estimated according to Fraser *et al.* [51]. VLDL particle volume was subsequently derived from its diameter. Apolipoprotein B (apoB) content of nascent VLDL particles was determined using Western Blot as previously described [52]. Four representative VLDL samples per group were analyzed and equal amounts of total lipid were loaded onto the gel. Signal intensity was quantified using a Molecular Imager (ChemiDoc XRS System, Bio-Rad Laboratories, Hercules, CA) and the relative abundance of apoB48 versus apoB100-associated particles was calculated.

### Statistics

All data are presented as means±SEM. Statistical analysis was performed using Brightstat software (www.brightstat.com). Analysis of two groups (chow *vs.* high-fat, high-fat *vs.* high-fat/fish oil) was assessed by Kruskal-Wallis using the Conover test for post-hoc analysis. Statistical significance was reached at a *p* value below 0.05.

## Supporting Information

Table S1 Primer and probe sequences used for quantitative PCR.
(0.03 MB DOC)
