# Supplementary material for: High Fat Feeding Induces Hepatic Fatty Acid Elongation in Mice
Source: PLoS One. 2009 Jun 26;4(6):e6066. doi: 10.1371/journal.pone.0006066 (PMC2699051; doi:10.1371/journal.pone.0006066)
Supplement: Table S1 — Primer and probe sequences used for quantitative PCR. (0.03 MB DOC) [file pone.0006066.s001.doc]

**Table S1. Primer and probe sequences used for quantitative PCR.**

| Gene | Sense | Antisense | Probe | Accession number |
| --- | --- | --- | --- | --- |
| *Elovl6* | ACA CGT AGC GAC TCC GAA GAT | AGC GCA GAA AAC AGG AAA GAC T | TTT CCT GCA TCC ATT GGA TGG CTT C | NM_130450.2 |
| *Hmgs (cyto)* | CGA TGG TGT AGA TGC TGG AAA G | CAT CAG TTT CTG AAC CAC AGT | CGA TCC GTG CAG AAG CCC ATC C | NM_145942.2 |
| *Pgc-1β* | GAG ACA CAG ATG AAG ATC CAA GCT | CTT GCC AAG AGA GTC GCT TTG T | CCA GGT GCC TCA TGC TGG CCT | NM_133249 |

*Elovl6*, fatty acid elongase 6; *Hmgs* *(cyto)*, 3-hydroxy-3-methylglutaryl-Coenzyme A synthase 1; *Pgc-1β*, peroxisome proliferator activated receptor gamma co-activator 1 beta.
